# Spike-induced cytoarchitectonic changes in epileptic human cortex are reduced via MAP2K inhibition

**DOI:** 10.1093/braincomms/fcae152

**Published:** 2024-04-29

**Authors:** Rachael A Smith, Fozia Mir, Mitchell P Butler, Biswajit Maharathi, Jeffrey A Loeb

**Affiliations:** Department of Neurology and Rehabilitation, University of Illinois Chicago, Chicago, IL 60612, USA; Department of Neurology and Rehabilitation, University of Illinois Chicago, Chicago, IL 60612, USA; Department of Neurology and Rehabilitation, University of Illinois Chicago, Chicago, IL 60612, USA; Department of Neurology and Rehabilitation, University of Illinois Chicago, Chicago, IL 60612, USA; Department of Neurology and Rehabilitation, University of Illinois Chicago, Chicago, IL 60612, USA

**Keywords:** neocortical epilepsy, cortical spiking, MEK, tetanus toxin, spatial memory

## Abstract

Interictal spikes are electroencephalographic discharges that occur at or near brain regions that produce epileptic seizures. While their role in generating seizures is not well understood, spikes have profound effects on cognition and behaviour, depending on where and when they occur. We previously demonstrated that spiking areas of human neocortex show sustained MAPK activation in superficial cortical Layers I–III and are associated with microlesions in deeper cortical areas characterized by reduced neuronal nuclear protein staining and increased microglial infiltration. Based on these findings, we chose to investigate additional neuronal populations within microlesions, specifically inhibitory interneurons. Additionally, we hypothesized that spiking would be sufficient to induce similar cytoarchitectonic changes within the rat cortex and that inhibition of MAPK signalling, using a MAP2K inhibitor, would not only inhibit spike formation but also reduce these cytoarchitectonic changes and improve behavioural outcomes. To test these hypotheses, we analysed tissue samples from 16 patients with intractable epilepsy who required cortical resections. We also utilized a tetanus toxin-induced animal model of interictal spiking, designed to produce spikes without seizures in male Sprague–Dawley rats. Rats were fitted with epidural electrodes, to permit EEG recording for the duration of the study, and automated algorithms were implemented to quantify spikes. After 6 months, animals were sacrificed to assess the effects of chronic spiking on cortical cytoarchitecture. Here, we show that microlesions may promote excitability due to a significant reduction of inhibitory neurons that could be responsible for promoting interictal spikes in superficial layers. Similarly, we found that the induction of epileptic spikes in the rat model produced analogous changes, including reduced neuronal nuclear protein, calbindin and parvalbumin-positive neurons and increased microglia, suggesting that spikes are sufficient for inducing these cytoarchitectonic changes in humans. Finally, we implicated MAPK signalling as a driving force producing these pathological changes. Using CI-1040 to inhibit MAP2K, both acutely and after spikes developed, resulting in fewer interictal spikes, reduced microglial activation and less inhibitory neuron loss. Treated animals had significantly fewer high-amplitude, short-duration spikes, which correlated with improved spatial memory performance on the Barnes maze. Together, our results provide evidence for a cytoarchitectonic pathogenesis underlying epileptic cortex, which can be ameliorated through both early and delayed MAP2K inhibition. These findings highlight the potential role for CI-1040 as a pharmacological treatment that could prevent the development of epileptic activity and reduce cognitive impairment in both patients with epilepsy and those with non-epileptic spike-associated neurobehavioural disorders.

## Teaser

Smith *et al*. identify a loss of inhibitory interneurons in high-spiking areas of epileptic human cortex. In a rat model of spiking, MAP2K inhibition reduces spikes and minimizes interneuron loss and microglial activation. This work highlights novel therapeutic targets for epilepsy, attention-deficit hyperactivity disorder and traumatic brain injury.

## Graphical Abstract

**Graphical Abstract:**
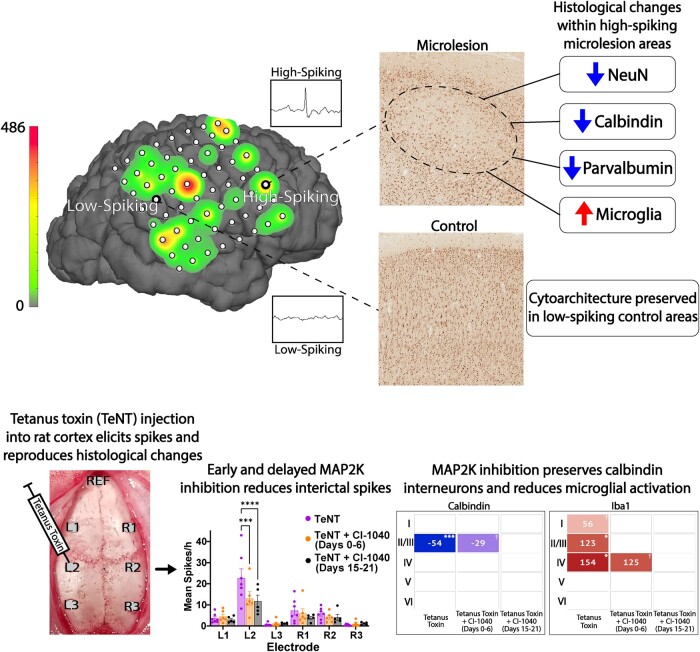

## Introduction

Epilepsy is a common neurological disorder defined by spontaneous seizures and associated with extensive behavioural comorbidities. Interictal epileptiform discharges, or interictal spikes, are frequently observed in patients with epilepsy. Spikes are brief (<200 ms) paroxysmal discharges that are generated over large brain regions and detected using EEG.^1^ Over 90% of people with epilepsy have interictal spikes detected across repeated EEGs.^2,3^ Following a brain injury, spikes may develop prior to seizures, suggesting that they could promote seizure development.^4,5^ This argument is strengthened by the finding that removal of high-spiking areas correlates with improved surgical outcomes.^6^ Not all spikes are associated with seizures; spikes have been recorded from patients with neurobehavioural disorders including anxiety, attention-deficit hyperactivity disorder (ADHD), autism spectrum disorder (ASD), obsessive-compulsive disorder, depression and schizoaffective disorder.^7-12^ Between 5% and 60% of patients with ADHD and up to 70% of patients with ASD have spike activity on EEG, raising the possibility that spikes could contribute to both cognitive and behavioural disorders.^12-15^

Not surprisingly, patients with epilepsy experience cognitive and behavioural comorbidities that have been associated with interictal spikes: up to 50% of spikes may result in transitory cognitive impairment.^16,17^ In adults with epilepsy, spikes impair memory maintenance and word retrieval, delay reaction times, disrupt executive functioning and increase the risk of crashing a car in a virtual driving simulation.^18-21^ Furthermore, neuronal synchrony induced by persistent spiking can disrupt cortical function in areas distant to the spike onset region, resulting in widespread cognitive deficits induced by localized spiking.^22,23^ Thus, spikes could serve as a biomarker for cognitive impairment in adults with epilepsy.

In animal models, spikes impair short-term memory, spatial memory and object recognition.^24,25^ Spikes differentially affect behaviours depending on which brain regions they involve. We previously found that locomotor activity is strongly correlated with spike location.^26^ Additionally, spikes during early life can have pronounced effects into adulthood; when induced in rat pups, spikes were associated with impaired spatial memory performance and reduced long-term potentiation in adult animals.^27^ Early spikes also cause deficits in sociability and attention that persist into adulthood, suggesting interventions to reduce spikes during childhood could have long-lasting benefits.^28,29^ Currently, there are no therapeutics specifically designed to prevent or treat epileptic spiking.

Understanding the aetiology of interictal spikes has proven challenging. In patients with epilepsy, spikes occur more frequently than seizures but often occur in the same brain regions, making it difficult to disentangle the pathophysiology of spikes from that of seizures. We curated an extensive collection of human cortical tissues, collected during resective epilepsy surgeries, where each sample is precisely mapped with long-term intracranial EEG *in vivo* recordings. Transcriptional comparisons of high- and low-spiking cortical regions show highly consistent changes including activation of the MAPK and CREB pathways in cortical Layers I–III.^30-32^ MAPK signalling has an activity-dependent component, with calcium signalling initiating the MAPK cascade.^33^ Increased neuronal activity near high-spiking areas causes persistent MAPK activation leading to increased CREB signalling, increased neuronal excitability and strengthened synaptic connections between high-spiking neurons.^34-37^ Within areas of increased MAPK activity, we also identified ‘microlesions’: areas with a marked reduction in neuronal (NeuN) staining and increased microglial activation centred in deeper cortical layers.^30,31^ Although nuclear NeuN staining was reduced, neuronal cell bodies were preserved within microlesion areas. Additionally, the presence of microlesions was significantly correlated with spike frequency in epileptic human neocortex.^31^

To investigate the molecular underpinnings of spiking, we optimized a rat model of interictal spiking induced with tetanus toxin (TeNT) injection into Layer II of the somatosensory cortex.^38,39^ This model reliably produces spontaneous epileptic spiking without seizures to permit the analysis of spikes independent from seizures. It also replicates the activation of CREB and downstream transcriptional changes seen in human cortex.^39^ We previously showed that early inhibition of MAPK using a MAP2K (also known as MEK) inhibitor blocks CREB activation and significantly reduces interictal spiking in rats.^39^

Here, we characterize the cytoarchitectonic changes in human epileptic spiking-associated microlesions, revealing a reduction of inhibitory interneurons that may lead to large-scale neuronal synchrony. These changes are reproducible in our rat model of interictal spiking. Using the lipophilic MAP2K inhibitor, CI-1040, we can block spike development, reduce epileptic spiking across time and minimize spatial memory impairments. CI-1040 has already advanced to Phase II clinical trials as an anti-cancer agent, with few adverse effects reported, and could be readily repurposed to prevent the development of interictal spikes following brain injury.^40,41^

## Materials and methods

### Human tissue collection

Human neocortical tissue samples were obtained from 16 patients who underwent surgery for drug-resistant epilepsy. Patients were enrolled following informed consent as approved by an Institutional Review Board at Wayne State University (IRB #2015–0457). Only brain tissues that would otherwise be discarded were used, and no additional tissue was removed beyond what was indicated for seizure treatment. Tissues were subsequently transferred to the University of Illinois at Chicago NeuroRepository. Patient demographics are provided in Supplementary Table 1. Patients underwent a two-stage surgery with long-term subdural electrocorticography (ECoG) and at least 3 days of continuous *in vivo* monitoring to identify regions of seizure onset and interictal spiking, which were then removed during the second stage of the procedure.^31,42,43^ Tissue was divided into 1 cm^3^ samples corresponding to a precise brain location overlying a single ECoG electrode. Quantitative measures of electrical activity were calculated as the number of spikes in 10 min averaged from three independent 10 min recordings using an automatic algorithm described previously.^31,44,45^ Tissue samples were stained with Luxol fast blue and haematoxylin and eosin and reviewed by a neuropathologist to confirm that they were devoid of histopathological abnormalities or surgical haemorrhage.

### Tissue processing

For immunohistochemistry, tissue samples corresponding to precise electrode locations were fixed with 4% paraformaldehyde, cryoprotected in 30% sucrose and embedded in OCT (TissueTek) before mounting as described previously.^30^ The 20 µm sections were prepared and stored frozen until use. Immunohistochemistry was performed using VECTASTAIN® Elite® ABC Universal PLUS Kit (#PK-8200, Vector Labs). Tissue samples were probed with the following antibodies: NeuN (1:4000, #MAB337, Millipore), Iba-1 (1:1000, #019-019741, Wako), calbindin (CB) (1:800, #131767, Cell Signaling Technologies), somatostatin (SST) (1:50, #ab111912, Abcam) and parvalbumin (PV) (1:4000, #ab11427, Abcam).

### Image analysis of human histological sections

Slides were imaged using an Aperio AT2 microscope system (Leica). Images were exported in a .tif format for subsequent analysis. For each sample section, we selected six regions of interest (ROIs) spanning cortical Layers I–VI, with a width of 1500 ± 100 pixels. Each ROI was subdivided into 10 equally sized horizontal layers representing the cortical layers. To quantify any changes in the microlesions as compared with control areas, each individual layer was analysed using ImageJ (Analyze Particles function).^46^ Per cent area stained (signal area divided by total area) was used to compare staining intensity. Data were exported into GraphPad Prism (version 9) for graphing and statistical analyses.

### Animals

Two-month-old male Sprague–Dawley rats were acquired from Envigo. Animals were housed in individual cages (to reduce the risk of premature headcap detachment) with *ad libitum* access to food and water throughout the study. Rats were entrained on a 12 h light–dark cycle with lights-on beginning at sunrise. Rats were sacrificed at 8 months of age. Additionally, three surgery-naïve male Sprague–Dawley rats were included to control for effects of epidural electrode placement. Rats were monitored daily by University of Illinois Chicago (UIC) veterinary care staff. All experimental protocols were approved by the UIC Animal Care Committee (ACC Protocol #21-081) and adhered to institutional guidelines.

### TeNT injection and intracranial electrode implantation surgery

Rats were anaesthetized with 5% isoflurane in 100% O_2_ (induction) followed by 2.5% isoflurane in 100% O_2_ (maintenance) at a rate of 2 L/min. The scalp was shaved and cleaned with iodine and isopropanol. A subcutaneous injection of 1% lidocaine (0.2 mL) and 0.125% bupivacaine (0.3 mL) was delivered under the scalp 10 min before the first incision. Rats were placed in a Kopf stereotaxic frame to stabilize the skull. A rectal temperature probe and infrared heating pad were used to monitor and maintain the animal’s temperature at 37°C throughout surgery. An incision was made from the midline of the skull above the nasal sinus to the posterior occiput (∼3 cm in length). The scalp was retracted and the periosteum was removed. The area was cleaned with 10% H_2_O_2_ and dried with sterile gauze. An electrocautery tool was used to achieve haemostasis along the skull surface. Seven holes were drilled into the skull surface using a 1/16 inch drill bit (three left and three right electrode holes: AP +4 mm, ML 3.5 mm; AP −1 mm, ML 3.5 mm; AP −6 mm, ML 3.5 mm; 1 reference electrode hole above the nasal sinus: AP +10.5 mm, ML 0.5 mm left), exposing the surface of the dura. A 1 µL Hamilton syringe attached to a micromanipulator was used to inject rats in the left somatosensory cortex at the L2 electrode hole (AP −1 mm, ML 3.5 mm left, DV −1.5 mm). Rats were randomly assigned to receive either TeNT or sham injections: TeNT-injected rats received 80 ng TeNT in 1 µL sterile PBS, and sham-injected rats received 1 µL sterile PBS. The injection was delivered over 4 min (0.25 µL/min), and the needle was left in place for 10 min to ensure localized toxin delivery. After removing the needle, seven customized epidural screw electrodes (3.2 mm wide, 7 mm long; P1 Technologies) were screwed into the skull to a depth of 1 mm and fixed to two six-channel plastic electrode pedestals (#MS363, P1 Technologies). A thin layer of Vetbond tissue adhesive (3 M) was applied to the surface of the skull to aid in headcap retention. Dental cement was applied around the electrode screws and pedestals to create a headcap apparatus. The scalp incision was closed with a metal wound clip, and antibiotic ointment was applied to the scalp margins. Rats were placed in a warming chamber set to 37°C and allowed to recover from anaesthesia for 1 h. Liquid acetaminophen (32 mg/mL) was added to the rats’ drinking water at a final concentration of 4 mg/mL to provide post-operative analgesia for 5 days following surgery.

### MAP2K inhibitor dosing and delivery

Rats are fitted with headcaps to enable EEG acquisition for the duration of our study. The rate of premature headcap detachment (a humane endpoint) is ∼20%, and excessive manipulation of the skull can increase headcap loss. To avoid drug delivery methods that require restraint, we optimized a voluntary oral delivery method.^47^ To acclimate rats to the oral drug delivery method, 2 g raw sugar cookie dough (Pillsbury) was provided to all rats at 0800 h each day for 5 consecutive days prior to surgery. Rats were randomly divided into three drug treatment groups: control, early drug dose and delayed drug dose [sham, *n* = 6; sham + CI-1040 Early, *n* = 6; sham + CI-1040 Delayed, *n* = 6; TeNT, *n* = 7; TeNT + CI-1040 Early, *n* = 8; TeNT + CI-1040 Delayed, *n* = 8]. Rats in the drug treatment groups received 250 mg/kg/day of the non-competitive MAP2K1/2 inhibitor, CI-1040 (MedChemExpress), delivered in 2 g raw cookie dough. This dose effectively reduced MAPK activation as shown in Supplementary Fig. 1. The early drug group received drug beginning on the day of surgery (first dose administered 2 h prior to surgery) and at 0800 h each day thereafter through post-operative Day 6; these rats also received 2 g cookie dough without drug on post-operative Days 15–21. Rats in the delayed drug group received 2 g cookie dough without drug on the day of surgery through post-operative Day 6 and cookie dough with drug from post-operative Days 15–21. The control group received 2 g raw cookie dough on the day of surgery through post-operative Day 6 and again on post-operative Days 15–21. All rats were monitored to ensure complete consumption.

### EEG and video recordings

EEG data were acquired at a sampling rate of 1000 Hz using Stellate HARMONIE software (version 6.0, Stellate Systems Inc.) and a time-locked video recording system. Continuous video EEG monitoring was carried out for up to 24 h per recording day. For EEG acquisition, rats were removed from their home cages and placed in individual acrylic recording cages with an EEG tether attached to the cage lid. Following attachment to the EEG tethers, animals were able to move freely throughout their recording cages. Rats received *ad libitum* access to food and water during recordings. EEG signal quality was assessed at the beginning of each recording by examining electrode impedance: electrodes with impedance exceeding 50 kΩ were excluded from subsequent analyses. EEG recordings occurred at the following intervals: every 5 days (beginning on post-operative Day 5 through post-operative Day 35), every 7 days (post-operative Days 35–84) and monthly (post-operative Day 84 through the 6-month study endpoint). Stellate EEG files were converted into European Data Format (EDF) files for subsequent visualization (EDFbrowser, version 1.76; Free Software Foundation) and data analysis. EDF files were filtered using a 1–35 Hz bandpass filter and a fourth-order Butterworth filter. All figures containing EEG data were generated using a referential montage in EDFbrowser. EDF files were manually reviewed for recording artefacts before implementing our automated spike detection algorithm.

### Barnes maze

To assess spatial memory function, Barnes maze testing was carried out using a custom-made 20-hole maze adapted from Rosenfeld *et al*.^48^ Testing was conducted over three 5-day trials: Trial 1 (pre-surgery), Trial 2 (post-operative Days 15–19) and Trial 3 (post-operative Days 56–60). On Day 1 of each trial, rats were acclimated to the Barnes maze layout and directed into the escape hole prior to testing. From Days 2–5, rats were allowed to explore the maze without being directed into the escape hole. Testing ended after the rat entered the escape hole or after 5 min elapsed. The test was recorded as a failure if the rat (i) did not enter the escape hole within the 5 min testing period or (ii) jumped or fell from the maze and was not captured and returned to the centre of the maze within 10 s. Between trials, the location of the escape hole was moved relative to a set of fixed visual cues within the testing room. Barnes maze testing was conducted between 0700 and 1100 daily, and rats were assigned to a random testing order each day. Each test was video recorded, and performance metrics were assessed with EthoVision XT software (version 16; Noldus).

### Tissue collection

In preparation for perfusion, rats received an overdose of isoflurane anaesthesia. Rats were perfused with 500 mL of heparinized PBS and subsequently decapitated. Brains were removed and divided into six coronal sections of either 2 or 3 mm thickness using a brain matrix. Sections were cut so that each pair of electrodes was bisected along the coronal plane. The posterior region was designated for histology (3-mm-thick section). Sections designated for histology were post-fixed for 48 h in 4% paraformaldehyde (PFA) in phosphate-buffered saline (PBS) and cryoprotected in 30% sucrose in Tris-buffered saline (TBS). Tissue was flash-frozen in OCT blocks, cryosectioned into 20-µm-thick sections and stored at −20°C prior to immunohistochemical staining.

### Immunohistochemistry

Tissue sections were washed in TBS and placed in blocking solution (0.05% Triton X-100, 5% goat or horse serum in TBS) for 1 h. Endogenous peroxidases were quenched with two 30 min incubations in a peroxide–sodium azide solution (0.1% sodium azide, 0.3% H_2_O_2_ in TBS). Tissue was placed in primary antibody (Iba-1: 1:750, #019-019741, Wako; CB: 1:150, #131767, Cell Signaling Technologies; SST: 1:50, #ab111912, Abcam; PV: 1: 4000, #ab11427, Abcam; NeuN: 1:4000, #MAB337, Millipore; GFAP: 1:800, #MAB360, Millipore) overnight at 4°C. Immunohistochemistry was performed using VECTASTAIN Elite ABC-HRP Kit [1:250, goat anti-rabbit (#PK-6101) or horse anti-mouse (#PK-6102)]. Images were acquired with an Aperio AT2 microscope (Leica) and exported in a .tif format for subsequent analysis in ImageJ.

### Image analysis of rat histological sections

A minimum of three animals from each treatment group and three naïve animals (no surgical or drug exposure) were included in the histological analysis (sham, *n* = 3; sham + CI-1040 early, *n* = 3; sham + CI-1040 Delayed, *n* = 3; TeNT, *n* = 4; TeNT + CI-1040 Early, *n* = 5; TeNT + CI-1040 Delayed, *n* = 5; naïve, *n* = 3]. Brightfield images were converted to greyscale and inverted in Adobe Photoshop. For each histological section, three ROIs in the left cortex were identified using ImageJ software. ROIs extended from the cortical surface to the corpus callosum and were oriented so that each ROI was perpendicular to the cortical surface. The height of each ROI varied based on the cortical thickness, and the width was maintained at 1500 ± 100 pixels. ROI thresholding was performed by a blinded observer using ImageJ. ROIs were then subdivided into 10 equally sized horizontal layers, based on the initial image height, with each subdivision representing approximately the same cortical depth across all ROIs. The Analyze Particles function in ImageJ (with a particle size threshold of 100–2000 pixels^2^) was used to quantify the signal for each ROI. Per cent area stained (signal area divided by total area) was used to compare signal intensity across regions. For each animal, we calculated the mean per cent area stained within each cortical layer across all three ROIs.

### Statistical analysis

We conducted an *a priori* power analysis using existing spike data for sham and TeNT-injected rats.^39^ To achieve a power > 0.80 with an alpha value of 0.05 and an estimated Cohen’s *d* of 1.36, we required a minimum of four animals per experimental group. Additionally, we estimated a 20% rate of premature headcap loss and included two additional animals per group. Rats were assigned to groups using a random sequence generator. Investigators were blinded to experimental groups during data analysis, and automated algorithms were used to mitigate bias when quantifying spikes. For histological analysis of human cortex, two-way ANOVA or mixed effects analysis with Šidák’s multiple comparisons test was used to compare control versus microlesion areas. For histological analysis of rat cortex, two-way ANOVAs with Tukey–Kramer multiple comparisons tests were used to identify significant differences between surgical (sham versus TeNT) groups or within surgical groups and between drug treatment groups. When comparing individual values for mean spikes/h, robust regression and outlier removal (ROUT) analysis (*Q* = 1%) was used to identify high-spiking outliers within each group (Supplementary Fig. 2). Prior to outlier removal, there were no significant differences between in spiking between the TeNT only and TeNT + drug treatment groups (TeNT versus TeNT + CI-1040 Early, *P* = 0.99; TeNT versus TeNT + CI-1040 Delayed, *P* = 0.97) or the TeNT Early versus Delayed groups (*P* = 0.82). These results were driven by several animals with extremely high spike frequencies (ROUT identified outliers) likely reflecting a Type II error. Outliers were excluded from subsequent drug efficacy analyses but included in grouped behavioural analyses to elucidate relationships between excessive spiking and behaviour. To analyse the relationship between Barnes maze outcomes and spiking, we performed a linear regression analysis and calculated Pearson’s correlation coefficient. Statistical analysis was performed using R and GraphPad Prism.^49^ A *P*-value cut-off of <0.05 was considered statistically significant. All data are presented as mean ± SEM. Figures were constructed using GraphPad Prism, EDFbrowser, Adobe Illustrator and the ggplot2 package in R.

## Results

### Microlesions in spiking human neocortex have reduced inhibitory interneurons and increased microglia

**Figure 1:**
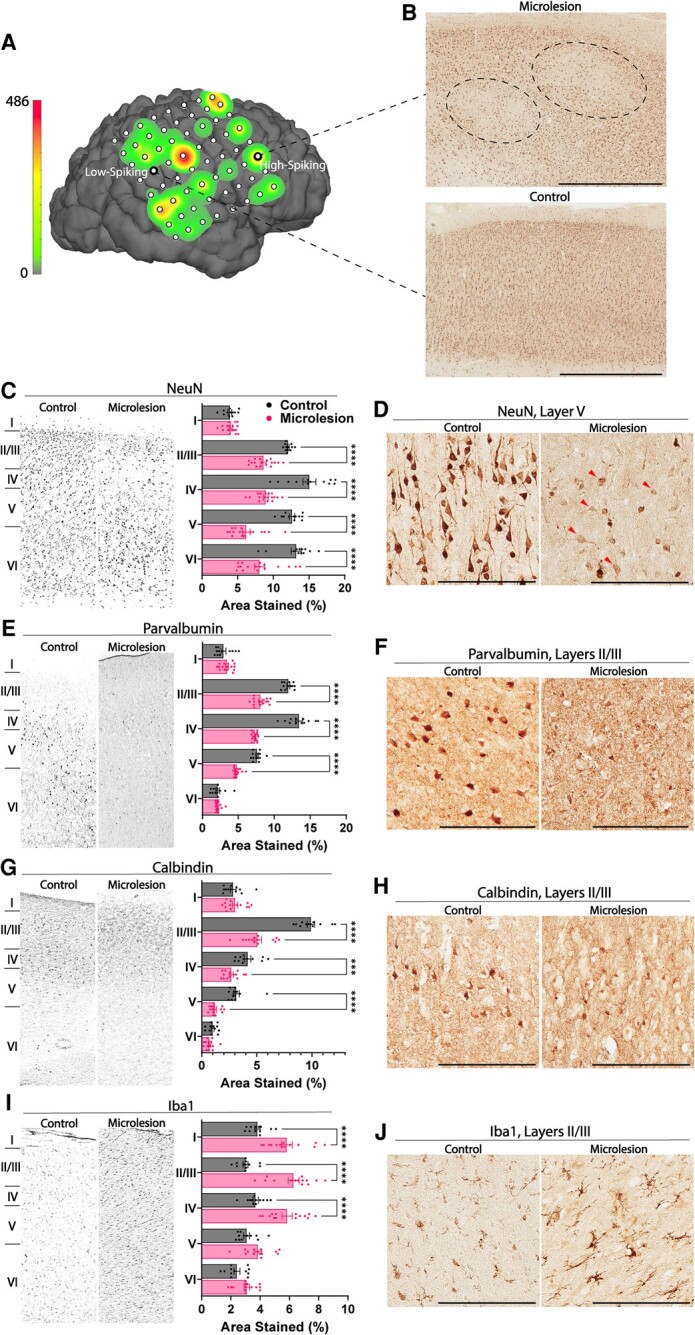
**High-spiking areas of human neocortex have fewer neuronal nuclear protein (NeuN)-positive neurons and inhibitory interneurons along with increased microglia.** (**A**) Long-term subdural electrodes are used to identify areas that produce seizures and spikes before surgical resection. An example of interictal spike frequencies, for one patient, at individual electrode locations is shown as a three-dimensional heatmap. Relative spike frequencies for each region are represented as green and red for low- and high-spiking areas, respectively. The relationship between relative spike frequency and colour scale is illustrated to the left of the brain schematic, with the maximum spike frequency reaching 486 spikes per 10 min recording segment for a single electrode. (**B**) Low- and non-spiking areas are used as internal controls and have few areas of NeuN signal loss and microlesions compared with high-spiking cortical regions. Scale bar: 2 mm. (**C**) Microlesion areas have significantly reduced NeuN staining in cortical Layers II–VI (*F*(1, 135) = 171.9, *P* < 0.0001). Control, *n* = 12; microlesion, *n* = 17. (**D**) Neurons with reduced NeuN staining (red arrowheads) are most frequently observed within Layer V of microlesions. (**E**) Parvalbumin (PV) interneurons are significantly reduced in Layers II–V of high-spiking cortex (*F*(1, 130) = 278.8, *P* < 0.0001). Control, *n* = 12; microlesion, *n* = 16. (**F**) PV reduction is most visually prominent in cortical Layers II and III of microlesions. (**G**) Calbindin (CB) staining is also reduced in Layers II–V of high-spiking cortex compared with control cortex. Control, *n* = 12; microlesion, *n* = 14. (**H**) CB reduction is most prominent in Layers II and III of microlesions. (**I**) High-spiking areas have increased Iba1 (microglial) staining in cortical Layers I–IV (*F*(1, 120) = 109.0, *P* < 0.0001). Control, *n* = 12; microlesion, *n* = 14. (**J**) Microlesions have larger microglia with more processes, most notably in cortical Layers II and III. Panels **D**, **F**, **H** and **J** taken at ×20 magnification. Scale bars represent 200 µm. ***P < 0.001, ****P < 0.0001 using two-way ANOVA or mixed effects analysis with Šidák’s correction for multiple comparisons. Error bars: ±SEM.

Twenty-nine samples, devoid of other pathologies, from high-spiking, low-spiking and non-spiking cortical brain regions from 16 different patients (Supplementary Table 1) were selected as shown in Fig. 1A. Each sample was precisely mapped to long-term intracranial electrical brain recordings as described.^43^ High-spiking samples had significantly more microlesions with reduced NeuN staining compared with low-/non-spiking control samples (Fig. 1B–D). This corresponds with our previously published findings showing that microlesions occurred more frequently in high-spiking areas and that the loss of nuclear NeuN within microlesions is not associated with a reduction in the number of neurons.^31^

Cortical layer-specific quantitative analysis of NeuN staining showed a significant reduction in Layers II–VI in high-spiking samples compared with control brain regions, with the greatest decrease observed in Layer V (Fig. 1C and D). These same layers showed a significant reduction in inhibitory interneurons. PV interneurons were decreased in Layers II–V of high-spiking human cortex as compared with low-/non-spiking cortex, with Layer IV showing the largest reduction in PV staining (Fig. 1E and F). CB interneurons were also reduced in microlesion areas compared with low-/non-spiking regions, with the greatest loss occurring in Layers II and III (Fig. 1G and H). Microlesions also had increased microglia across cortical Layers I–IV (Fig. 1I and J). These results suggest that cortical epileptic spiking could result from a reduction in inhibitory interneurons in deeper cortical layers leading to increased neuronal synchrony, excitability and connectivity in superficial Layers I–III.^30^

### Epileptic spiking in rodents replicates cytoarchitectonic changes seen in human spiking neocortex

**Figure 2:**
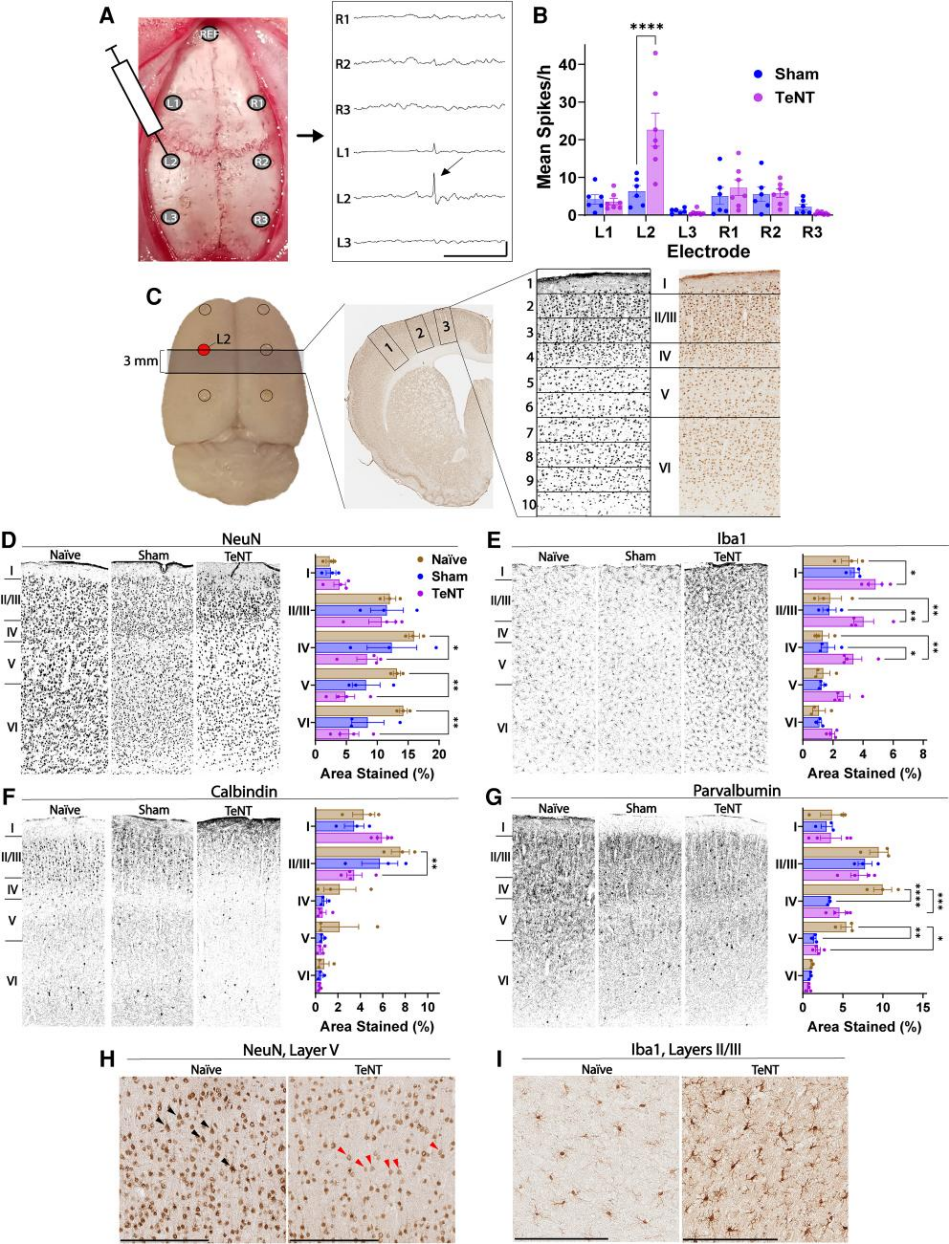
**Tetanus toxin (TeNT)-induced spiking causes cytoarchitectonic changes in rat cortex that recapitulate those observed in high-spiking areas of human cortex.** (**A**) Spikes at the injection site (L2 electrode) have a unique short-duration and high-amplitude morphology. Scale bars: 0.5 s (horizontal) × 1000 µV (vertical). (**B**) Compared with sham animals, TeNT-injected rats had a significant increase in mean spikes/hour (across all EEG recording days) at the L2 electrode (*F*(5, 168) = 7.017, *P* < 0.0001). Subsequent histochemical analyses targeted this region. Sham, *n* = 6; TeNT, *n* = 7 (**C**) Three mm coronal sections of cortex, bisecting the L2 electrode site, were collected for immunohistochemical staining. Brightfield images of the left hemisphere were divided into 3 cortical regions, which were extracted and subdivided into 10 horizontal regions of interest of equal size, corresponding to cortical Layers I–VI. For each animal, and each cortical layer, one mean value was calculated across all three regions to provide a global measure of cell loss within that cortical layer. (**D**) TeNT rats have a loss of neuronal nuclear protein (NeuN)-positive neurons relative to surgically naïve (control) rats (*F*(2, 35) = 9.351, *P* = 0.0006). TeNT rats have a significant reduction in NeuN signal in Layers IV–VI. Naive, *n* = 3; sham, *n* = 3; TeNT, *n* = 4. (**E**) Compared with both sham and naïve rats, TeNT rats had increased Iba1 (microglia) staining that was most pronounced in cortical Layers II and III but extended from Layers I–IV (*F*(2, 35) = 21.07, *P* < 0.0001). Naive, *n* = 3; sham, *n* = 3; TeNT, *n* = 4. (**F**) TeNT rats had a loss of calbindin (CB) staining in Layers II/III relative to naïve rats (*F*(2, 35) = 3.764, *P* = 0.0331). Naive, *n* = 3; sham, *n* = 3; TeNT, *n* = 4. (**G**) Sham and TeNT animals had a loss of parvalbumin (PV) signal in cortical Layers IV and V, with the greatest loss in Layer IV (*F*(2, 35) = 14.69, *P* < 0.0001). Naive, *n* = 3; sham, *n* = 3; TeNT, *n* = 4. (**H**) NeuN loss is most pronounced in the neuronal nuclei within Layer V (×20 magnification). Black arrowheads, neurons with fully stained nuclei; red arrowheads, neurons with diminished nuclear NeuN staining. (**I**) Microglia from TeNT animals had more processes and were more densely packed than those in naïve animals (×20 magnification). Scale bars represent 200 µm. ­*P < 0.05, **P < 0.01, ***P < 0.001, ****P < 0.0001 using two-way ANOVAs with Tukey–Kramer *post hoc* tests. Error bars: ±SEM.

TeNT was injected into the left somatosensory cortex, corresponding to the L2 electrode site (Fig. 2A), resulting in focal epileptic spikes not seen in sham (saline-injected) animals (Fig. 2B). Video and EEG data were manually reviewed for all animals to confirm that spikes occurred in the absence of seizures. High-spiking cortical regions were subsequently probed for neuronal and microglial markers (Fig. 2C). Neuronal NeuN staining was significantly reduced within the left cortex of the TeNT animals compared with surgically naïve controls, with the greatest reduction in Layers V and VI (Fig. 2D). There were no significant differences in the number of neuronal cell bodies within Layer V. Paralleling our findings in spiking human cortex, TeNT-injected spiking animals also had increased microglial staining across Layers I–IV of the left cortex relative to both sham and naïve animals, with the greatest increase in Layers II–IV (Fig. 2E). In these same spiking regions, TeNT animals also had a loss of inhibitory interneurons relative to naïve and sham animals. For CB interneurons, TeNT animals had significant reduction in staining in Layers II/III (Fig. 2F). Interestingly, for PV interneurons, both sham and TeNT animals had a loss of staining in cortical Layers IV and V (Fig. 2G), suggesting that these neurons may be sensitive to even low levels of spiking activity. Closer histological analysis of NeuN staining in naïve and TeNT animals showed that cytoplasmic NeuN signal persisted within neurons, but nuclear NeuN was almost completely lost (Fig. 2H). A similar comparison of Iba1 histology showed that TeNT animals had an increase in the density of microglia and the number of microglial processes (Fig. 2I). We previously showed that there were no significant differences in gene expression or histology for astroglial markers between high- and low-spiking areas of human tissue.^31^ Similarly, there were no significant differences in GFAP staining in rat tissue (Supplementary Fig. 3). SST staining was also unaffected by spiking in both human and rat tissue (Supplementary Fig. 3).

### Early and delayed treatment with CI-1040 reduces interictal spiking

Given that MAPK activation occurs in spiking human and rat cortex^30,39^ and that MAP2K inhibition can significantly prevent the development of epileptic spiking when delivered acutely,^39^ we examined the effect of MAP2K inhibition at both early (0–7 days after TeNT injection) and delayed timepoints (15–21 days after TeNT injection) to assess the effects of MAP2K inhibition after spikes have already developed. To confirm drug target engagement, a 7-day oral treatment with 250 mg/kg/day of the MAP2K1/2 inhibitor, CI-1040, led to a significant reduction in cytoplasmic diphosphorylated MAPK1/2 (also known as diphosphorylated ERK1/2) in the rat brain (*P* = 0.0052) (Supplementary Fig. 1) and a decrease in nuclear diphosphorylated MAPK1/2 (*P* = 0.0595). This demonstrates the ability of CI-1040 to cross the blood–brain barrier and inhibit MAPK activity within the brain. As discussed in the Statistical analysis section, ROUT analysis identified six high-spiking outliers (Supplementary Fig. 2) that were excluded from between-group analyses of drug or toxin effects. Final sample sizes for between-group analyses of drug effects were as follows: sham, *n* = 6; sham + CI-1040 Early, *n* = 5; sham + CI-1040 Delayed, *n* = 5; TeNT, *n* = 7; TeNT + CI-1040 Early, *n* = 6; TeNT + CI-1040 Delayed, *n* = 5.

**Figure 3:**
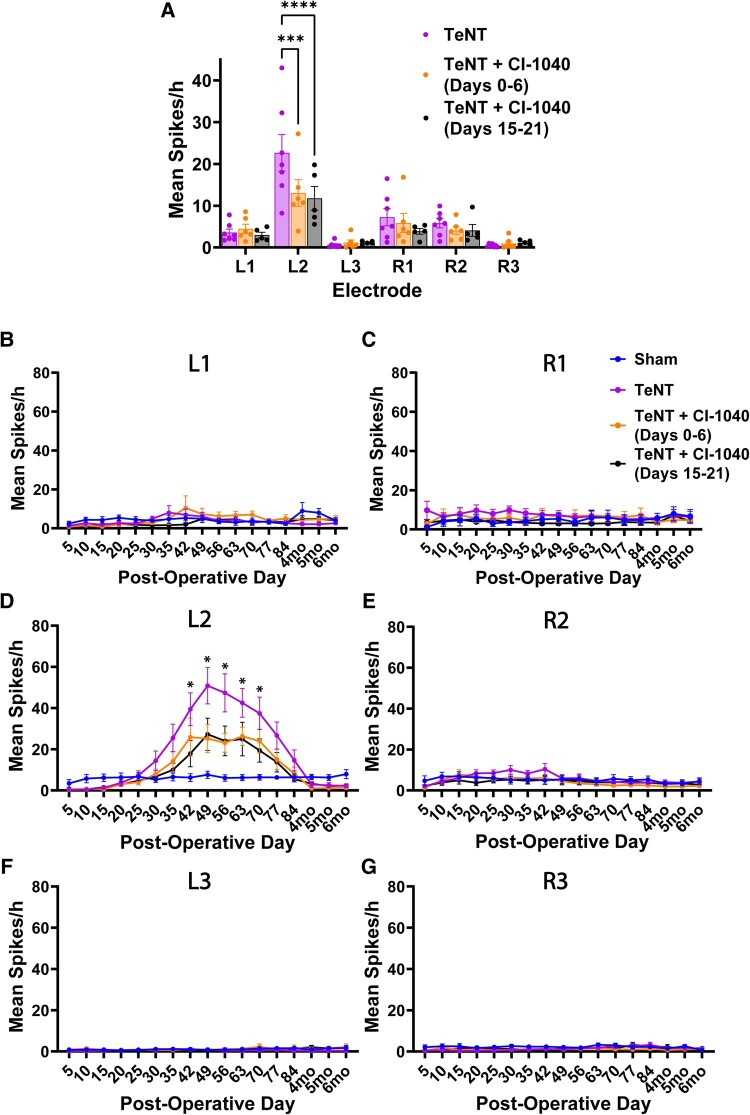
**Both early and delayed treatment with CI-1040 significantly reduce interictal spikes.** (**A**) Both early and delayed CI-1040 drug treatments significantly reduced the mean spikes/hour (across all EEG recording days) at the L2 electrode (*F*(5, 168) = 7.017, *P* < 0.0001). Tetanus toxin (TeNT): *n* = 7; TeNT + CI-1040 (Days 0–6): *n* = 6; TeNT + CI-1040 (Days 15–21): *n* = 5. (**B** and **C**) Low-level spiking was present across all groups in the anterior electrodes. (**D**) In TeNT-injected rats, spikes began to increase above sham levels at Day 30, with maximum spiking occurring at Day 49, followed by a steady decline in spiking that returned to sham levels by 4 months post-surgery (*F*(5, 28) = 6.164, *P* = 0.0006). (**E**) Low levels of spiking were also seen at the R2 electrode, contralateral to the TeNT injection site, across all groups. (**F** and **G**) The posterior electrodes showed almost no spikes in any group. L1, left anterior electrode; L2, left middle electrode and saline/TeNT injection site; L3, left posterior electrode; R1, right anterior electrode; R2, right middle electrode; R3, right posterior electrode. For panels **B**–**G**: sham, *n* = 6; TeNT, *n* = 7; TeNT + CI-1040 (Days 0–6), *n* = 6; TeNT + CI-1040 (Days 15–21), *n* = 5. *P < 0.05, ***P < 0.001, ****P < 0.0001 using two-way repeated measures ANOVAs or mixed effects analysis with Tukey–Kramer *post hoc* tests. Error bars: ±SEM.

TeNT-injected animals had the highest spike frequency at the L2 electrode, corresponding to the toxin injection site (Fig. 3A). Placement of electrodes in the sham-injected animals produced minimal spiking that was evenly distributed across the L1, L2, R1 and R2 electrodes (Fig. 3B–E). In both groups, spikes were rarely observed on the posterior L3 and R3 electrodes (Fig. 3F and G). Repeated measures two-way ANOVA of mean spikes/hour by electrode showed a significant reduction in spikes in the TeNT + CI-1040 early (*P* = 0.0001) and delayed groups (*P* < 0.0001) relative to the untreated TeNT group at the L2 electrode, suggesting that MAP2K inhibition can reduce spiking both acutely (concurrent with TeNT injection) and after spikes have started to develop (Fig. 3A).

Examination of mean spikes/hour on each recording day revealed three phases of spike development in TeNT animals: an acute phase of spike development (Month 1), a subacute phase of rapidly increasing spike frequency at the L2 injection site (Months 2–3) and a delayed phase where spike frequency returned to sham levels (Months 4–6). A mixed effects analysis of mean spikes/hour per recording day on the L2 electrode showed a significant increase in spike frequency within the TeNT group compared with the sham group at post-operative Days 42 (*P* = 0.0392), 49 (*P* = 0.0181), 56 (*P* = 0.0291) and 63 (*P* = 0.00132). There was also a significant increase in spike frequency in the TeNT + CI-1040 Early group compared with the sham group at Day 63 (*P* = 0.0331). Both early and delayed CI-1040 treatment blunted the peak number of mean spikes/hour from post-operative Days 42–63 (Fig. 3D).

### Early and delayed treatment with CI-1040 reduces interneuron loss and microglial activation

**Figure 4:**
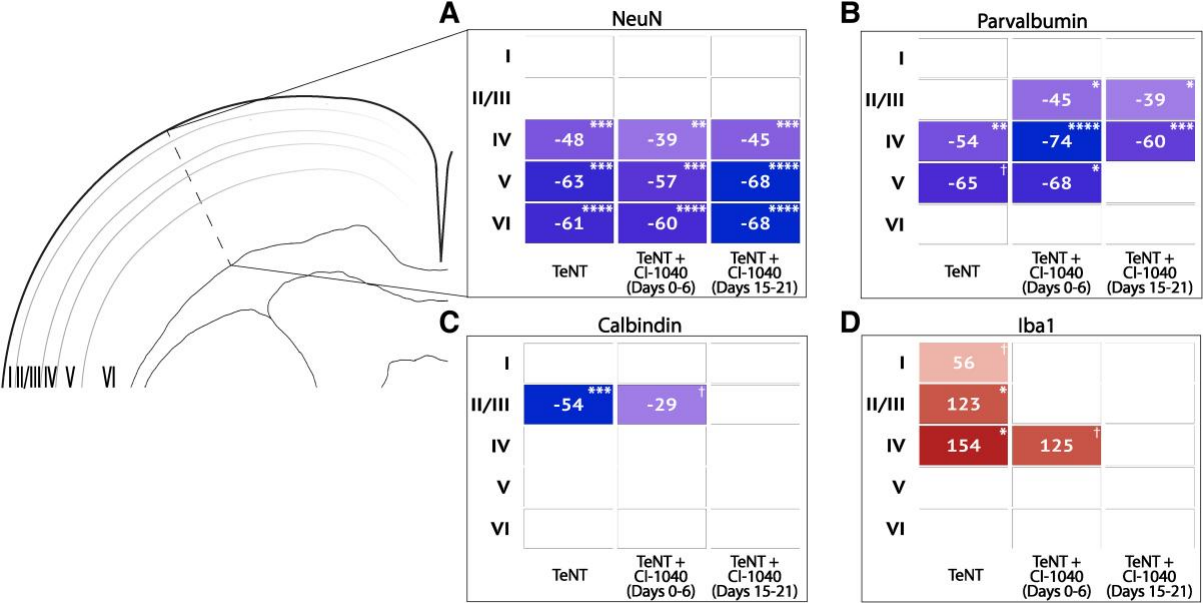
**Treatment with CI-1040 preserves cortical cytoarchitecture, increasing calbindin interneuron staining and reducing microglial activation in TeNT animals.** (**A**) The mean per cent area stained per cortical layer for each tetanus toxin (TeNT) group was evaluated as a per cent change relative to the naïve group. White areas indicate no significant difference between TeNT and naïve, blue areas indicate a significant reduction in TeNT animals, and red areas indicate a significant increase in TeNT animals. When compared with naïve animals, all TeNT groups, including those with CI-1040 treatment, had a significant reduction in neuronal nuclear protein (NeuN) staining (*F*(3, 65) = 19.21, *P* < 0.0001). (**B**) Neither early nor delayed CI-1040 treatment mitigated parvalbumin (PV) interneuron loss in Layer IV, and all groups had significantly less PV staining compared with naïve animals (*F*(3, 65) = 11.13, *P* < 0.0001). Interestingly, both early and delayed drug treatments reduced PV staining in Layers II/III, a finding not observed in non-drug-treated animals. (**C**) Early drug treatment reduced calbindin (CB) interneuron loss in Layers II and III by 46%, and delayed drug treatment further restored CB interneurons to the level of naïve animals (*F*(3, 65) = 3.088, *P* = 0.0332). (**D**) Early drug treatment normalized Iba1 (microglia) to naïve levels in Layers I–III and reduced microglial staining in Layer IV by 19%. Delayed drug treatment normalized microglia to the level of naïve animals across all cortical layers (*F*(3, 65) = 9.447, *P* < 0.0001). TeNT, *n* = 4; TeNT + CI-1040 (Days 0–6), *n* = 5; TeNT + CI-1040 (Days 15–21), *n* = 5. Analysis performed using two-way ANOVA with Tukey–Kramer *post hoc* tests. †*P* < 0.1, *P < 0.05, **P < 0.01, ***P < 0.001, ****P < 0.001.

CI-1040 treatment did not rescue NeuN or PV staining in TeNT animals (Fig. 4A and B; Supplementary Fig. 4); however, both early and delayed CI-1040 treatment significantly reduced the loss of CB staining in Layers II and III of the left cortex of TeNT animals, the region where CB staining was lowest within untreated TeNT animals (Fig. 4C). Delayed drug treatment in TeNT animals significantly reduced Iba1 staining relative to untreated animals, and early drug treatment caused a trending reduction in Iba1 staining in cortical Layer IV (*P* = 0.0775) (Fig. 4D). In contrast, early drug-treated sham animals had significantly less NeuN staining compared with naïve animals and significantly more Iba1 staining compared with non-drug sham and naïve animals (Supplementary Fig. 5).

### Spikes in TeNT-injected rats are morphologically distinct and highly sensitive to CI-1040 treatment

**Figure 5:**
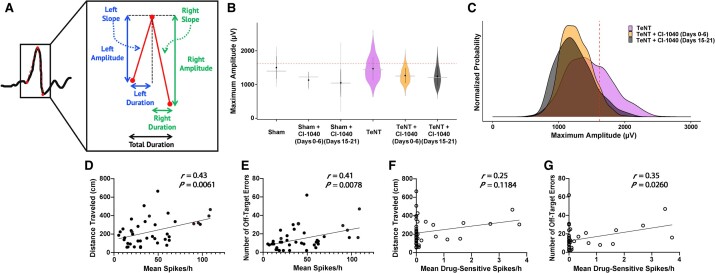
**CI-1040 alters spike morphology, which correlates with improved spatial memory performance on the Barnes maze test.** (**A**) For each spike, we measured left and right half-wave spike amplitudes and durations, slopes and total spike duration. (**B**) Among spikes which met the slope cut-off (>71 µV/ms), those with maximum amplitude at least 1 σ above the mean across all tetanus toxin (TeNT) groups (>1617 µV, red dashed line) were specific to TeNT animals. (**C**) Maximum amplitude distributions shown for all TeNT animals (regardless of treatment) demonstrated that both early and delayed MEK inhibition led to fewer spikes exceeding the maximum amplitude cut-off (red dashed line). (**D** and **E**) Spike frequency on post-operative Day 63 was significantly correlated with total distance travelled as well as off-target errors on the Barnes maze across all rats, regardless of treatment group. (**F**) Among drug-sensitive spikes (slope > 71 µV/ms, amplitude > 1617 µV), there was a trending correlation between increased spiking and increased distance travelled on the final day of Barnes maze Trial 3. (**G**) An increase in drug-sensitive spikes was significantly correlated with the number of off-target errors on the final day of Barnes maze Trial 3. *n* = 40 for each scatter plot. Sham, *n* = 6; sham + CI-1040 (Days 0–6), *n* = 5; sham + CI-1040 (Days 15–21), *n* = 5; TeNT, *n* = 7; TeNT + CI-1040 (Days 0–6), *n* = 6; TeNT + CI-1040 (Days 15–21), *n* = 5. For panels **D**–**G**, significance was assessed as *P* < 0.05 using linear regression with Pearson’s correlation (*r*).

Spikes were subclassified based on their morphology. Each waveform was approximated as a triangle from which the amplitude, duration and slope in both the first half (left) and second half (right) could be computed (Fig. 5A). We identified a population of spikes on the L2 electrode that distinguished TeNT-injected animals from sham animals (see Supplemental Methods). Specifically, spikes with a second-half slope of at least 71 µV/ms (slope cut-off) were observed in all animals injected with TeNT but present in only half of the sham animals. These spikes accounted for a larger fraction of the total spike count in TeNT animals compared with shams (1.36% versus 0.284%). Similar results were observed when comparing TeNT and sham animals treated with CI-1040. TeNT animals in both early and delayed drug treatment groups showed greater percentages of spikes above the slope cut-off than the corresponding sham groups [Early: 1.18% (TeNT) versus 0.19% (sham); Delayed: 1.36% (TeNT) versus 0.537% (sham)]. These TeNT-specific spikes were predominantly observed at the L2 injection site, with peak spiking occurring on Day 70, nearly 1 month after the overall spike peak at Day 49. Spikes above the slope cut-off had both shorter duration and higher amplitude than spikes below the cut-off.

Within this group of TeNT-specific spikes, we found a subpopulation of spikes that responded to CI-1040 treatment (Fig. 5B). These drug-sensitive spikes had a maximum amplitude of at least 1617 µV (amplitude cut-off), which was 1 SD above the mean value for all TeNT spikes (across all treatment groups) that exceeded the slope cut-off (see Supplemental Methods and Supplementary Fig. 6). CI-1040 treatment reduced spikes above the amplitude cut-off in both early and delayed drug treatment groups, which showed narrower distributions of spike amplitudes (Fig. 5C).

### Drug-sensitive spikes are correlated with impaired spatial memory performance

To understand the relationship between increased spiking and behavioural performance, we correlated the mean spikes/hour on post-operative Day 63, the EEG recording day closest to the final day of Barnes maze Trial 3, with Barnes maze performance outcomes across all groups. Here, we included high-spiking outliers to further elucidate the effects of increased spiking on behaviour. There was a significant correlation between increased spiking and increased distance travelled across all animals on the final day of Barnes maze Trial 3 (Fig. 5D). Similarly, increased spiking was correlated with an increased number of off-target errors, defined as exploration of any maze hole other than the escape hole (Fig. 5E). We then asked whether drug-sensitive spikes (second-half slope > 71 µV/ms, maximum amplitude > 1617 µV) also correlated with Barnes maze performance. The relationship between increased drug-sensitive spiking and increased distance travelled was not significant (Fig. 5F), but there was a significant correlation between drug-sensitive spikes and off-target errors: more spikes correlated with poorer performance (Fig. 5G). There were no significant differences in Barnes maze performance across time between surgical groups or drug treatment groups.

## Discussion

An imbalance in neuronal excitation is thought to underlie the pathophysiology of spiking and epilepsy. Here, we show for the first time that high-spiking human neocortex has fewer inhibitory CB and PV interneurons in deeper cortical layers with an increase in surrounding microglia. PV interneurons are responsible for regulating the cortical excitation–inhibition balance, and patients with temporal lobe epilepsy have fewer PV and CB interneurons within the hippocampus.^50,51^ A loss of PV interneurons has also been seen in patients with ASD and schizophrenia, while a reduction in CB interneurons is associated with cognitive decline, suggesting that these interneurons could play a critical role in the development of spike-associated cognitive and behavioural impairments.^51,52^ Although the role of microglia in epilepsy has been debated, several studies found that increased microglia are associated with worsened seizure outcomes via increased synaptic pruning of neuronal arbors and subsequent disruption of the excitation–inhibition balance.^53,54^ Our results align with these findings and suggest that a loss of inhibitory tone within deeper cortical layers, due to microglial pruning of inhibitory synapses, could contribute to the hyperexcitable state of pyramidal neurons in Layers II and III and promote continued spiking.

By inducing spikes in our animal model, through local injection of TeNT into the somatosensory cortex, we were able to replicate the histological changes observed in high-spiking human neocortex including a loss of nuclear NeuN staining, a reduction in PV and CB inhibitory interneurons and an increase in surrounding microglia. Previous studies have shown that intracortical injection of low-dose TeNT alone causes minimal neuronal loss and does not induce long-term microglial activation.^55-58^ These results suggest that continuous epileptic spiking is sufficient to induce and maintain these cytoarchitectonic changes. Interestingly, variations in the number of PV or CB interneurons can affect locomotor activity in rats, a parameter that we have shown to be altered in high-spiking animals.^26,59^ Furthermore, a paucity of NeuN staining has been documented following traumatic brain injury (TBI) in humans, suggesting that the cellular changes we observed may be an early sign of spike-induced cortical damage.^60^

Our previous functional genomic analysis suggested that spikes are chronically maintained through sustained MAPK signalling within superficial cortical Layers I–III in both human and rodent cortex.^30,39^ While we previously showed that early MAP2K inhibition prevents epileptic spiking,^39^ here, we added a delayed treatment window where MAP2K inhibition was shown to reduce spiking after spikes had already formed. This shows that MAP2K inhibition reduces spiking without simply suppressing TeNT activity, as TeNT is degraded and no longer active within the brain during the delayed treatment window.^61^ MAP2K inhibition most likely reduces spiking by blocking the development of hypersynchronous spike networks through inhibition of long-term potentiation.^62,63^ In addition to reducing spikes, CI-1040 also mitigated spike-associated neuroinflammation and inhibitory neuronal loss in TeNT animals, reducing the density of microglia and preserving CB interneurons in Layers II and III. Interestingly, rats in the delayed drug group had a slightly greater reduction in mean spikes/hour at the toxin injection site compared with animals in the early drug group (Fig. 3A), and their CB and microglial histology was similar to that of naïve animals (Fig. 4C and D). Thus, spiking may progress over time to synchronize larger neuronal populations through ongoing MAPK-dependent mechanisms. These findings suggest that CI-1040 could be used acutely to prevent or reduce cytoarchitectonic changes in other spike-associated pathologies, such as those that occur after a TBI.^64,65^

We also found that spike morphology was an important consideration. In TeNT-injected animals, a population (0.57% of total spikes) of short-duration, high-amplitude spikes were directly correlated with spatial memory impairments. Poor spatial memory function has previously been observed in animals with hippocampal spikes.^24^ Our results provide the first evidence that neocortical spikes have comparable effects on spatial memory. Similar high-amplitude, steeply sloped spikes have also been observed in patients with epilepsy.^66^ Anti-seizure medications (ASMs) reduce both seizures and spikes, but there is limited information on how specific types of spikes (location or morphology) respond to drug treatment. Treatment with CI-1040 was most effective at targeting these epileptic-like spikes, suggesting that spike morphology could be used to identify ‘druggable spikes’ that may have negative effects on cognitive function. This finding could be used to improve patient outcomes: delivering personalized medicine based on the types of spikes on each patient’s EEG.

An additional benefit of CI-1040 is that it is highly lipophilic, allowing greater brain penetration relative to less lipophilic MAP2K inhibitors. CI-1040 also has a large therapeutic index and is relatively well-tolerated, making it an ideal drug for reducing spikes in patients with epilepsy.^40^ Furthermore, CI-1040 may improve cognitive measures and memory performance in patients with spike-associated behavioural impairments such as ASD, ADHD and schizoaffective disorder, offering a novel treatment for patients without the side-effects associated with existing ASMs.

Our study had several limitations. For the human data, we previously showed that gene expression is differentially affected at areas of spike onset, spread or reverberation.^67^ Because spikes are inherently heterogeneous between individuals, we chose to focus on relative spike frequency within each patient. Moving forward, it will be necessary to perform a large-scale analysis of spike dynamics and their spatial relationship to cellular changes. For the animal data, all rats were sacrificed at the 6-month study endpoint, preventing us from assessing spike-induced histological changes across time. This can be addressed in future studies by including additional cohorts with earlier endpoints to understand how neuronal and glial populations change as spike networks develop. Next, there is some evidence that MAP2K inhibition can effectively reduce seizure activity,^68,69^ but it is unclear whether early spike inhibition will have a similar effect. Our current model was designed to elicit spikes alone, and future studies will require a larger dose of TeNT to induce both spikes and seizures. Furthermore, the spatial resolution afforded by our six-electrode array makes it challenging to identify areas of spike initiation or propagation, which may explain some of the variability that we observed between animals. Finally, we found that spike frequency began to decrease around 3 months regardless of drug treatment group. By 4 months, TeNT and sham animals had similar spike frequencies. This was an unexpected finding that likely reflected the low dose of TeNT selected to elicit spikes without seizures. Even after spike levels normalized, the effects of spiking on cortical cytoarchitecture persisted, suggesting that transient spiking can have long-lasting effects.

In conclusion, we found that spiking significantly alters microglia and neuronal populations within the cortex. Treatment with the MAP2K inhibitor, CI-1040, reduced epileptogenic spiking, reduced CB interneuron loss and microglial activation and was correlated with improved spatial memory performance. CI-1040’s favourable efficacy-to-toxicity ratio, good brain penetration and previous advancement to Phase II clinical trials suggest that it could be readily adapted as novel therapeutic to treat spike-associated cognitive dysfunction. Additionally, pulsed treatment with CI-1040 may reduce the development of spiking following a wide array of brain insults including trauma and stroke.

## Supplementary Material

fcae152_Supplementary_Data

## Data Availability

The data that support the findings of this study are openly available in Dryad at https://doi.org/10.5061/dryad.hx3ffbgm0 and within the Supplementary material.
