# Supplementary material for: Spike-induced cytoarchitectonic changes in epileptic human cortex are reduced via MAP2K inhibition
Source: Brain Commun. 2024 Apr 29;6(3):fcae152. doi: 10.1093/braincomms/fcae152 (PMC11089420; doi:10.1093/braincomms/fcae152)
Supplement: fcae152_Supplementary_Data [file fcae152_supplementary_data.docx]

**Supplementary Material**

**This file contains:**

**Supplementary Methods.**

**Supplementary Figure 1.** A seven-day oral dose of 250 mg/kg/day of CI-1040 significantly reduced cytoplasmic dpMAPK1/2 in the rat brain.

**Supplementary Figure 2.** Outlier analysis identified six non-responders within the drug treatment groups.

**Supplementary Figure 3.** Spiking does not affect somatostatin interneurons or astrocytes.

**Supplementary Figure 4.** CI-1040 increases calbindin staining and reduces microglial activation in TeNT animals.

**Supplementary Figure 5.** Early treatment with CI-1040 reduces NeuN and increases microglial staining in sham animals.

**Supplementary Figure 6.** Example cluster analysis of interictal spikes based on morphologic parameters.

**Supplementary Table 1.** Patient demographics for high- and low-spiking tissue samples

**Supplementary Methods**

**CI-1040 dose selection**

We conducted a 7-day dosing study to ensure that CI-1040 could cross the blood-brain barrier and effectively inhibit MAPK activity when delivered orally (Supplementary Fig. 1). Rats were divided into three groups: 7-days CI-1040 (*n* = 3), 7-days cookie dough (*n* = 3), or naïve (*n* = 3) (Supplementary Fig. 1A). Rats in the CI-1040 group received a dose of 250 mg/kg/day of CI-1040 in 2 g raw sugar cookie dough at 0800 h each day for seven consecutive days. Rats in the cookie dough group received 2 g raw cookie dough for seven consecutive days. Rats in the naïve group did not receive drug or cookie dough. Six hours after the final drug dose (or at 1400 h on day 7 for rats that did not receive drug) all rats were sacrificed via cardiac perfusion with heparinized PBS. Rats were decapitated and brains were collected. Each brain was bisected along the midline into left and right hemispheres. The left hemisphere was flash-frozen for subsequent homogenization and western blotting.

**Tissue homogenization and western blotting**

Fresh frozen brain tissue was added to a buffer solution (320 mM sucrose, 10 mM Tris-HCl, 1.7 mM sodium pyrophosphate, 10 mM NaF, 1 mM PMSF, 2 mM Na_3_VO_4_, Complete Mini Protease Inhibitor Cocktail (Roche, #11836153001; Basel, Switzerland‎)) and homogenized on ice using an immersion disperser (Kinematica AG; Malters, Switzerland). Tissue lysate was centrifuged at 2300 rpm for 10 min at 4°C. The pellet was collected and resuspended in sucrose-free buffer solution as the nuclear fraction. The supernatant was centrifuged again at 16,000 rpm for 1 h at 4°C. The supernatant was collected as the cytosolic fraction. Fractionated tissue lysate was stored at -80°C prior to western blotting.

Samples were prepared for western blotting by adding 20 µg of nuclear or cytoplasmic protein to Laemmli sample buffer (2x, BioRad, #1610737) and boiling at 95°C for 5 min. Prepared samples were loaded into a 10% TGX electrophoresis gel (BioRad, #456-1036) and run at 150 V for 1 h in Tris-Glycine-SDS buffer. Transfer components and PVDF membranes were equilibrated in a 20% methanol Tris-glycine buffer. Proteins were transferred at 100 V for 1 h and 15 min at 4°C. Membranes were removed from the transfer apparatus and placed in blocking solution (5% milk, 1% BSA, 0.1% TBST) for 1 h at room temperature, followed by overnight incubation at 4°C in primary antibody (Phospho-p44/42 MAPK1/2 1:1500; Cell Signaling Technologies, #4370S) diluted in blocking solution. Membranes were washed in TBST and incubated with secondary antibody (HRP-linked goat anti-rabbit 1:4000; BioRad, #170-6515) for 1 h at room temperature. ECL substrate (Pierce, #32106) was applied to the membranes for 1 min and membranes were placed into an autoradiography cassette. Membranes were exposed to film for 5 min (cytoplasmic fraction) or 10 min (nuclear fraction) and developed in an X-Omat film processer (Kodak; Rochester, NY). Membranes were stripped for 7 min with Restore PLUS Stripping Buffer (ThermoFisher, #46428) and re-blocked for 1 h at room temperature, followed by overnight incubation at 4°C in primary antibody (β-actin 1:3000; Cell Signaling Technologies, #3700S) diluted in blocking solution. Membranes were washed in TBST and incubated with secondary antibody (HRP-linked goat anti-mouse 1:4000; BioRad, #170-6516) for 1 h at room temperature. ECL substrate was applied to the membranes for 1 min and membranes were placed into an autoradiography cassette. Membranes were exposed to film for 5 min (cytoplasmic fraction) or 10 min (nuclear fraction) and developed in an X-Omat film processer. Films were digitally scanned and band intensities were quantified using ImageJ. Unpaired two-sided Student’s t-tests were used to compare normalized protein levels of diphosphorylated MAPK1/2 (dpMAPK1/2) in the cytoplasmic and nuclear fractions of rat brain homogenate (Supplementary Fig. 1B).

**Algorithm-based interictal spike detection**

To analyze EEG data for the presence of interictal spikes, we implemented a custom spike-detection algorithm developed using MATLAB software (R2021b; MathWorks). We adapted our spike algorithm from previously published versions to accommodate a 1000 Hz sampling rate and optimize calculation of spike morphologic parameters.^28,50^ Briefly, EDF files were imported into MATLAB using the *edfread* function. EDF files were filtered with a 1-35 Hz fourth-order Butterworth bandpass filter. Each electrode was analyzed for the presence of spikes; spikes were defined as high-amplitude events with negative polarity, shorter than 200 ms in duration, with a maximum voltage greater than one standard deviation above the median background signal. Both spikes (< 70 ms) and sharp waves (70-200 ms) were included in the overall spike count. Spikes were then divided into two half waves, for which amplitude, duration, and slope were calculated. Estimation of these metrics depends on accurate identification of the start and end points (edges) of each spike. These can be identified visually as the locations of the first trough points (local minima) that occur just before (start) and after (end) the point of maximum voltage. After identifying the spike location (point of maximum voltage), edge detection was performed in three essential steps. First, the raw signals were filtered using a fourth-order Butterworth highpass filter with 7 Hz cutoff frequency to eliminate the confounding effect of low-frequency background or prominent slow-waves. Next, the filtered signals were smoothed using a 12-point moving average window. Finally, the raw, filtered, and smoothed signals were inverted, and potential edge points were identified using the *findpeaks* function. True edges were designated as those which were detected consistently across the raw, filtered, and smoothed signals. Moreover, the maximum amplitude and total duration for each spike was determined. For each recording day, spike counts were normalized using the total recording length (mean spikes/hour). In addition to the spikes automatically detected by our algorithm, we also manually reviewed early EEG files (prior to post-operative day 30) to look for smaller spikes that the algorithm may have missed; however, for the purposes of this study we focused only on spikes that were detected by the algorithm to ensure an unbiased assessment of drug effects.

**Exploratory data analysis of spike morphology**

Exploratory data analysis was used to classify spikes based on individual morphology. Cluster analysis and plotting was performed in R using the tidyverse, factoextra, and gridExtra packages. We developed an R script which takes a dataframe with all spike morphology data as input and, leveraging a user-defined set of morphologic parameters, automatically implements k-means clustering and outputs plots like those shown in Supplementary Fig. 5. Morphologic data are analyzed and clustered independently for each experimental group. Briefly, the data are first scaled (i.e. converted to z-scores) to ensure that parameters with different ranges of values can be fairly compared. Next, outliers are removed via the built-in outlier detection in the R boxplot() function to avoid distorting the cluster analysis. Silhouette analysis [1,2] is then performed to determine the optimal number clusters, after which k-means clustering is performed using the the kmeans() function (stats package). Finally, the fviz_nbclust() function (factoextra package) is used to generate the annotated plots from the k-means clustering results. We tried various combinations of the fundamental spike morphologic parameters (i.e. Left Amplitude, Left Duration, Right Amplitude, Right Duration) computed by our spike detection algorithm. For simplicity, and since it provided the most clear and informative results, we have shown the iteration where we included only Right Amplitude and Right Duration (Supplementary Fig. 5).

We identified a population of spikes with a steep second-half slope that were seen across all animals injected with TeNT but were infrequent in sham animals. A specific cutoff value of 71 µV/ms (3.2σ cutoff) was obtained by: (1) computing an individual cutoff for both sham and TeNT animals (μ + 3.2σ) of the second-half slope of all spikes at the L2 electrode across treatment groups; and (2) taking the average of these two cutoffs. Using this second-half slope cutoff, we were able to separate spikes observed in TeNT-injected animals from those of sham-injected animals [number of spikes above the 3.2σ cutoff: TeNT: 1576 spikes, 1.73 % of total spikes; TeNT + CI-1040 Early: 671 spikes, 1.18 % of total spikes; TeNT + CI-1040 Delayed: 549 spikes, 1.36 % of total spikes; Sham: 138 spikes, 0.284 % of total spikes; Sham + CI-1040 Early: 39 spikes, 0.19 % of total spikes; Sham + CI-1040 Delayed:163 spikes, 0.537 % of total spikes]. These TeNT-specific spikes were predominantly observed at the L2 injection site, with peak spiking occurring on day 70, nearly one month after the overall spike peak at day 49. Spikes above the slope cutoff were both shorter in duration and higher in amplitude than spikes below the cutoff.

Within this group of TeNT-specific spikes, we found a subpopulation of spikes that responded to CI-1040 treatment. These drug-sensitive spikes had a maximum amplitude 1σ above the mean value for all TeNT spikes exceeding the slope threshold [amplitude cutoff = (((mean max. amplitude + 1σ of TeNT spikes > 71 µV/ms on L2) + (mean max. amplitude + 1σ TeNT Early spikes > 71 µV/ms on L2) + (mean max. amplitude + 1σ TeNT Delayed spikes > 71 µV/ms on L2))/3) = 1617 µV]. CI-1040 treatment reduced spikes above the amplitude threshold in both early and delayed drug treatment groups, narrowing the distribution of spike amplitudes below the cutoff value [number of spikes above the 1σ cutoff: TeNT: 516 spikes, 35% of all spikes > 71 µV/ms on L2; TeNT + CI-1040 Early: 47 spikes, 7.4% of all spikes > 71 µV/ms on L2; TeNT + CI-1040 Delayed: 51 spikes, 9.7% all spikes > 71 µV/ms on L2].


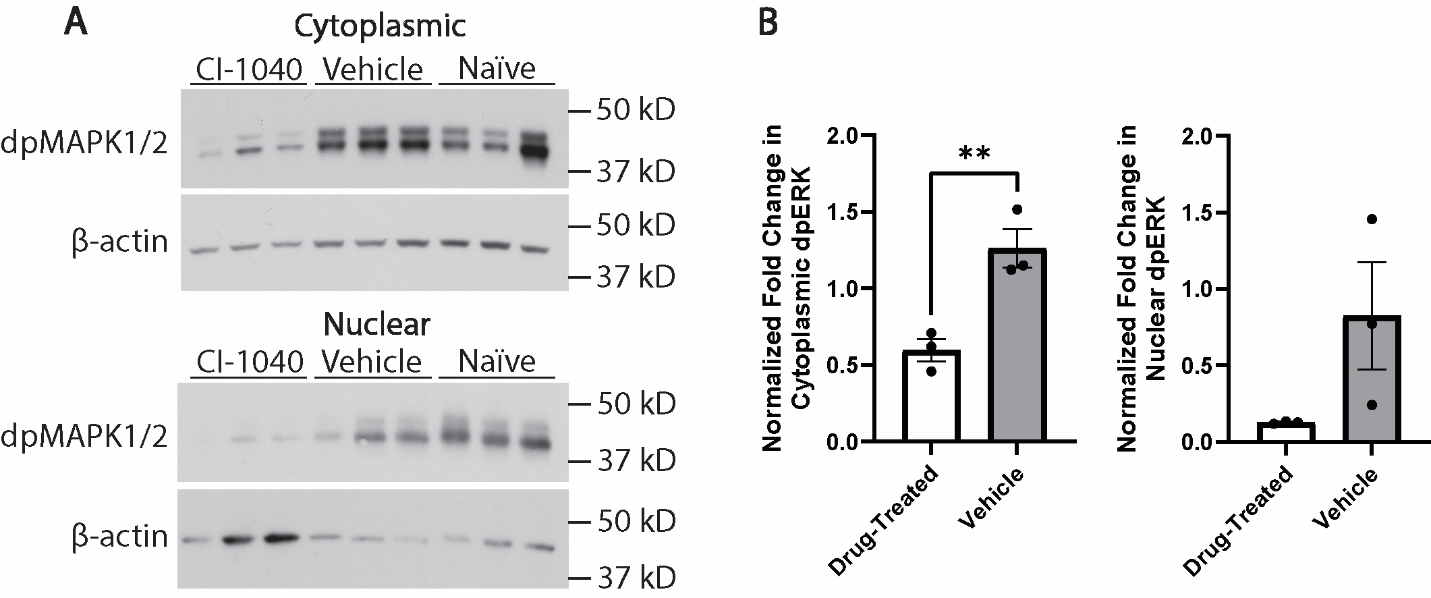
**Supplementary Figure 1 A seven-day oral dose of 250 mg/kg/day of CI-1040 significantly reduced cytoplasmic dpMAPK1/2 in the rat brain. (A)** Rats were assigned to one of three groups (CI-1040 treatment, vehicle only, or naïve control). Rats in the drug-treated group received a 7-day dose of 250 mg/kg/day CI-1040 delivered orally in 2 g raw sugar cookie dough. Vehicle-only rats received 2 g of cookie dough for 7 days. Naïve rats were maintained on a diet of *ad libitium* rat chow without supplementation. Rats were sacrificed 6 h after the final dose of drug or cookie dough and brains were collected and fractionated for western blotting. Each lane represents an individual rat. (**B)** Protein levels of nuclear and cytoplasmic dpMAPK1/2 were normalized by dividing the relative signal intensity of dpMAPK1/2 by β-actin for each lane. Fold change in dpMAPK1/2 protein levels for drug-treated and vehicle-only rats was calculated by dividing normalized dpMAPK1/2 values by the mean dpMAPK1/2 protein quantity for naïve rats. Treatment with CI-1040 significantly reduced cytoplasmic levels of dpMAPK1/2 (*t*(4) = 4.56, *P* = 0.0052) and caused a substantial reduction in nuclear dpMAPK1/2 (*t*(4) = 1.98, *P* = 0.0595) in the rat brain. ***P* < 0.01 using an unpaired two-sided Student’s t-test. Error bars represent ± SEM.


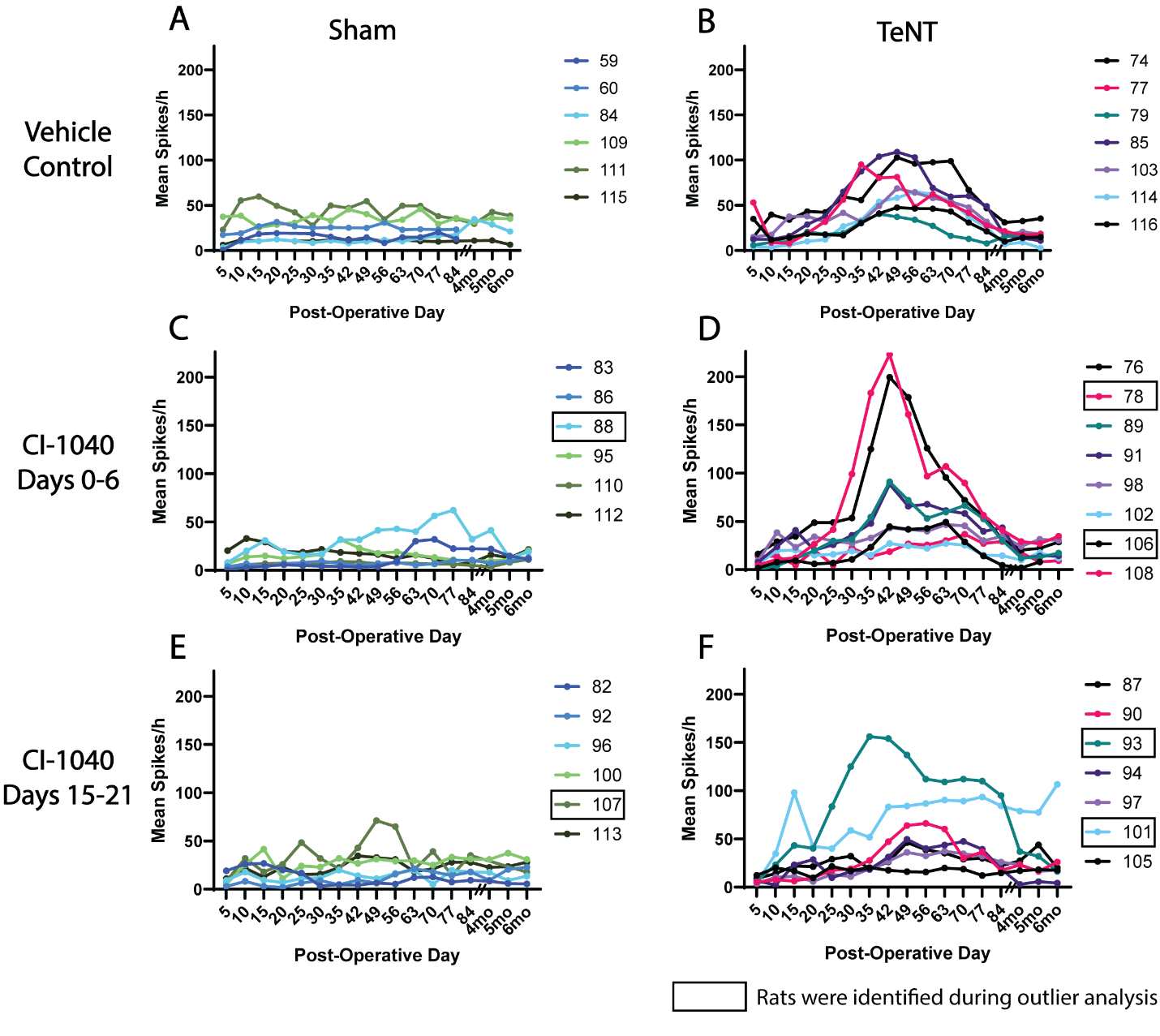
**Supplementary Figure 2** **Outlier analysis identified six non-responders within the drug treatment groups. (A-B)** There were no outliers within the vehicle-only sham or tetanus toxin (TeNT) groups. **(C)** Robust regression and outlier removal (ROUT) analysis identified one high-spiking outlier (animal 107) within the Sham + CI-1040 Early group. **(D)** Two outliers (animals 78 and 106) were identified in the TeNT + CI-1040 Early group. **(E)** One outlier (animal 107) was identified in the Sham + CI-1040 Delayed group. **(F)** Two outliers (animals 93 and 101) were identified in the TeNT + CI-1040 Delayed group. Outliers were excluded from between-group analyses of drug or toxin effects but were included in behavioral studies which considered all animals together, regardless of group. Figure legends represent individual animal numbers.


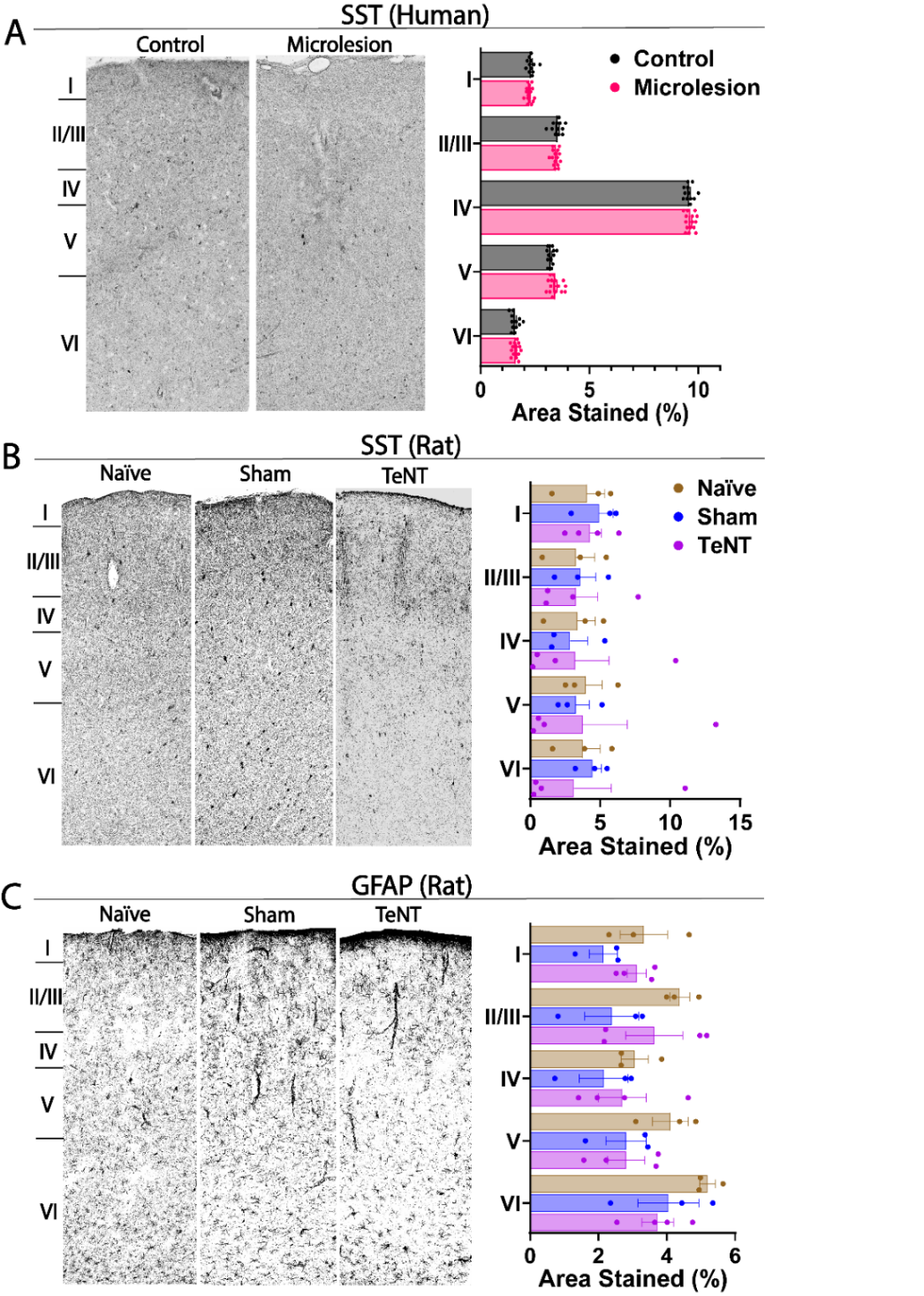
**Supplementary Figure 3 Spiking does not affect somatostatin interneurons or astrocytes. (A)** Histological analysis revealed no significant difference in somatostatin (SST) interneuron staining between control and microlesion areas of human cortex (*F*(1, 125) = 0.658, ns). Control: *n* = 12; Microlesion: *n* = 15. **(B)** Similar to the findings in human tissue, there are no significant differences in SST staining between surgically naïve (control), Sham, and tetanus toxin (TeNT) rats (*F*(2, 35) = 0.0299, ns). Naïve: *n* = 3; Sham: *n* = 3; TeNT: *n* = 4 **(C)** Staining for GFAP (astrocytes) revealed a significant group effect (*F*(2, 35) = 5.423, *P* = 0.0089); however, post hoc tests showed no significant differences between naïve, Sham, and TeNT rats. Naïve: *n* = 3; Sham: *n* = 3; TeNT: *n* = 4. Analysis performed using two-way ANOVA with Šidák’s or Tukey’s correction for multiple comparisons for human and rats, respectively. Error bars: ± SEM.


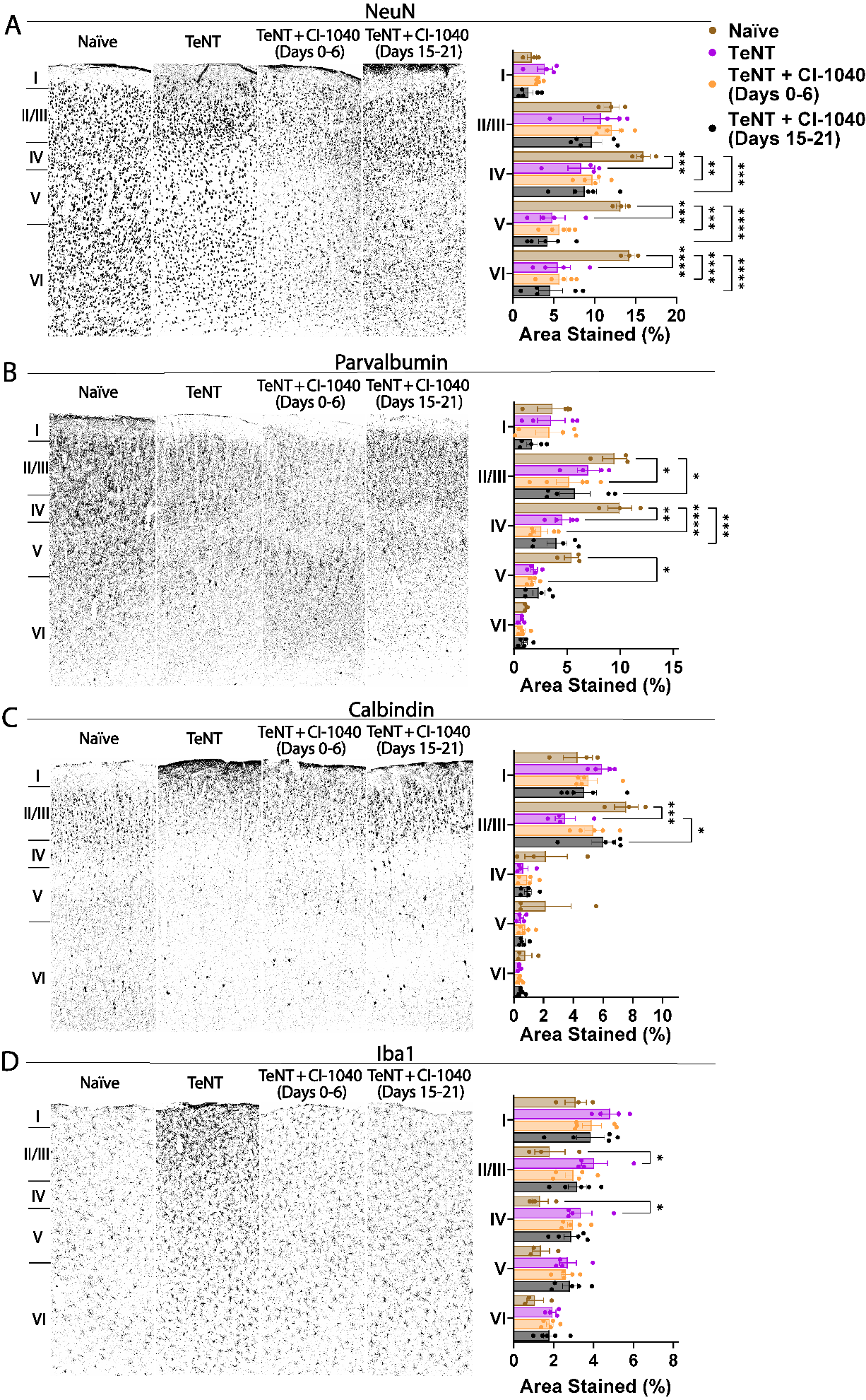


**Supplementary Figure 4 CI-1040 increases calbindin staining and reduces microglial activation in TeNT animals. (A)** There was a significant reduction in neuronal nuclear protein (NeuN) staining in layers IV-VI across all tetanus toxin (TeNT) groups, regardless of drug treatment (*F*(3, 65) = 19.21, *P* < 0.0001). **(B)** Both early and delayed CI-1040 treatment increased parvalbumin interneuron loss in layer IV, relative to untreated TeNT animals (*F*(3, 65) = 11.13, *P* < 0.0001). Early drug treatment also resulted in a significant reduction in parvalbumin interneuron loss in layer V, compared to naïve animals. Interestingly, both early and delayed drug treatments reduced parvalbumin staining in layers II/III, a finding not observed in the TeNT no drug group. **(C)** There was a significant group effect of CI-1040 treatment on calbindin staining (*F*(3, 65) = 3.088, *P* = 0.0332). TeNT animals in the drug treatment groups had similar levels of calbindin staining in layers II-III compared to naïve animals. Furthermore, delayed drug treatment significantly increased the level of calbindin staining compared to untreated TeNT animals. **(D)** While untreated TeNT animals had a significant increase in Iba1 staining in layers II-IV compared to naïve animals (*F*(3, 65) = 9.447, *P* < 0.0001), animals treated with CI-1040 had no significant differences in Iba1 staining compared to the naïve group. Naïve: *n* = 3; TeNT: *n* = 4; TeNT + CI-1040 (Days 0-6): *n* = 5; TeNT + CI-1040 (Days 15-21): *n* = 5. Analysis performed using two-way ANOVA with Tukey-Kramer post-hoc tests. **P* < 0.05, ***P* < 0.01, ****P* < 0.001, **** *P* < 0.0001.


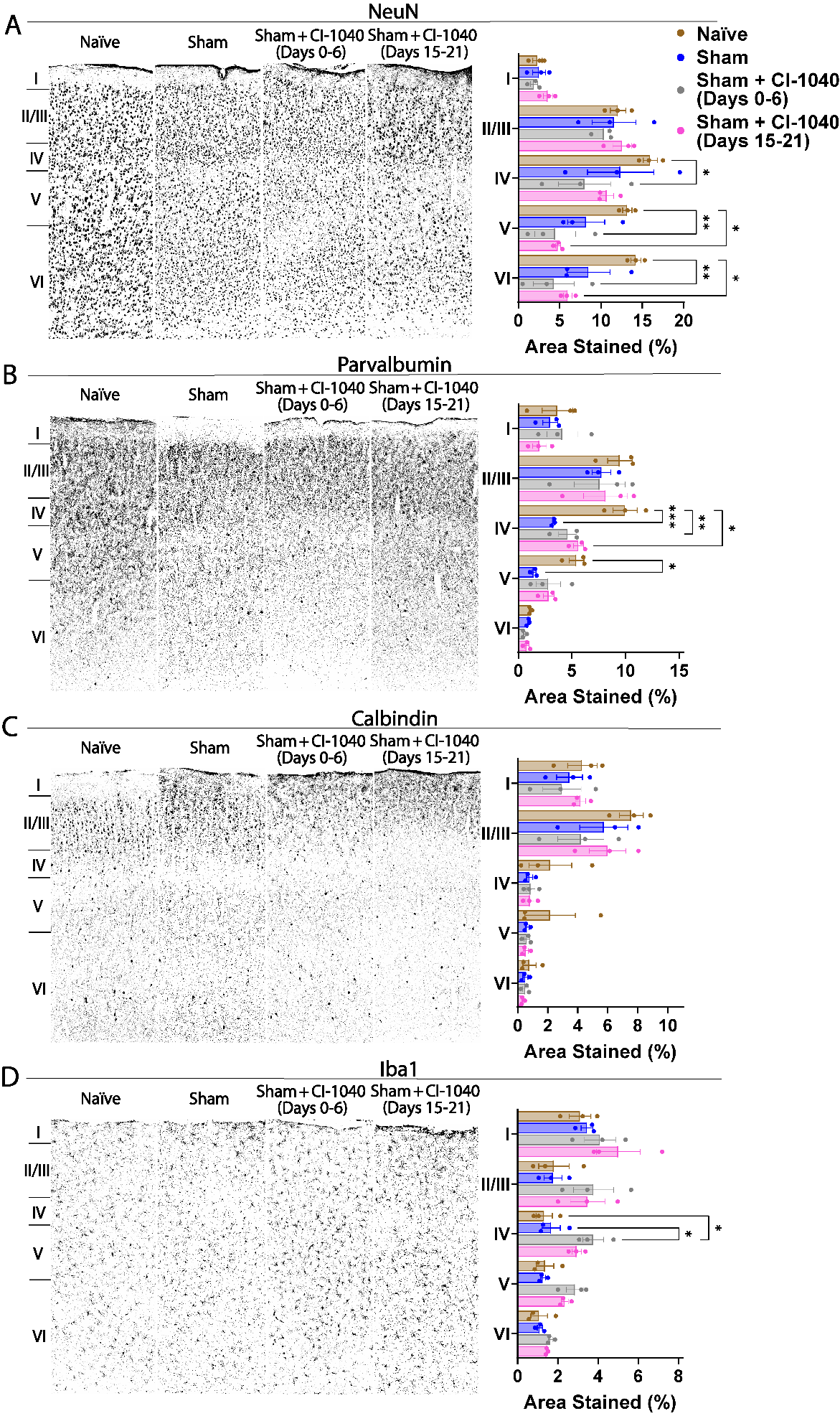


**Supplementary Figure 5 Early treatment with CI-1040 reduces NeuN and increases microglial staining in sham animals. (A)** There was a significant effect of drug treatment on neuronal nuclear protein (NeuN) staining (*F*(3, 40) = 9.133, *P* < 0.0001) such that animals in the Sham + CI-1040 Early group had a significant reduction in NeuN staining in layers IV-VI and animals in the Sham + CI-1040 Delayed group had a significant reduction in NeuN staining in layers V-VI. **(B)** Parvalbumin staining was significantly reduced across all sham groups compared to naïve animals (*F*(3, 40) = 6.239, *P* = 0.0014). Animals in the Sham no drug group had the largest reduction in parvalbumin staining in layers IV-V, while animals in the drug treatment groups had a smaller reduction in parvalbumin staining that was restricted to layer IV alone. **(C)** There were no significant differences between any of the sham groups and the naïve animals in calbindin staining (*F*(3, 40) = 2.789, ns). **(D)** There was a significant group effect on Iba1 staining (*F*(3, 40) = 10.19, *P* < 0.0001), with early drug treatment causing a significant increase in microglia in layer IV and a trending increase in layers II/III (*P* = 0.069). There was also a trending increase in microglia in layer I for animals in the delayed drug treatment group (*P* = 0.083). Naïve: *n* = 3; Sham: *n* = 3; Sham + CI-1040 (Days 0-6): *n* = 3; Sham + CI-1040 (Days 15-21): *n* = 3. Analysis performed using two-way ANOVA with Tukey-Kramer post-hoc tests. **P* < 0.05, ***P* < 0.01, ****P* < 0.001.


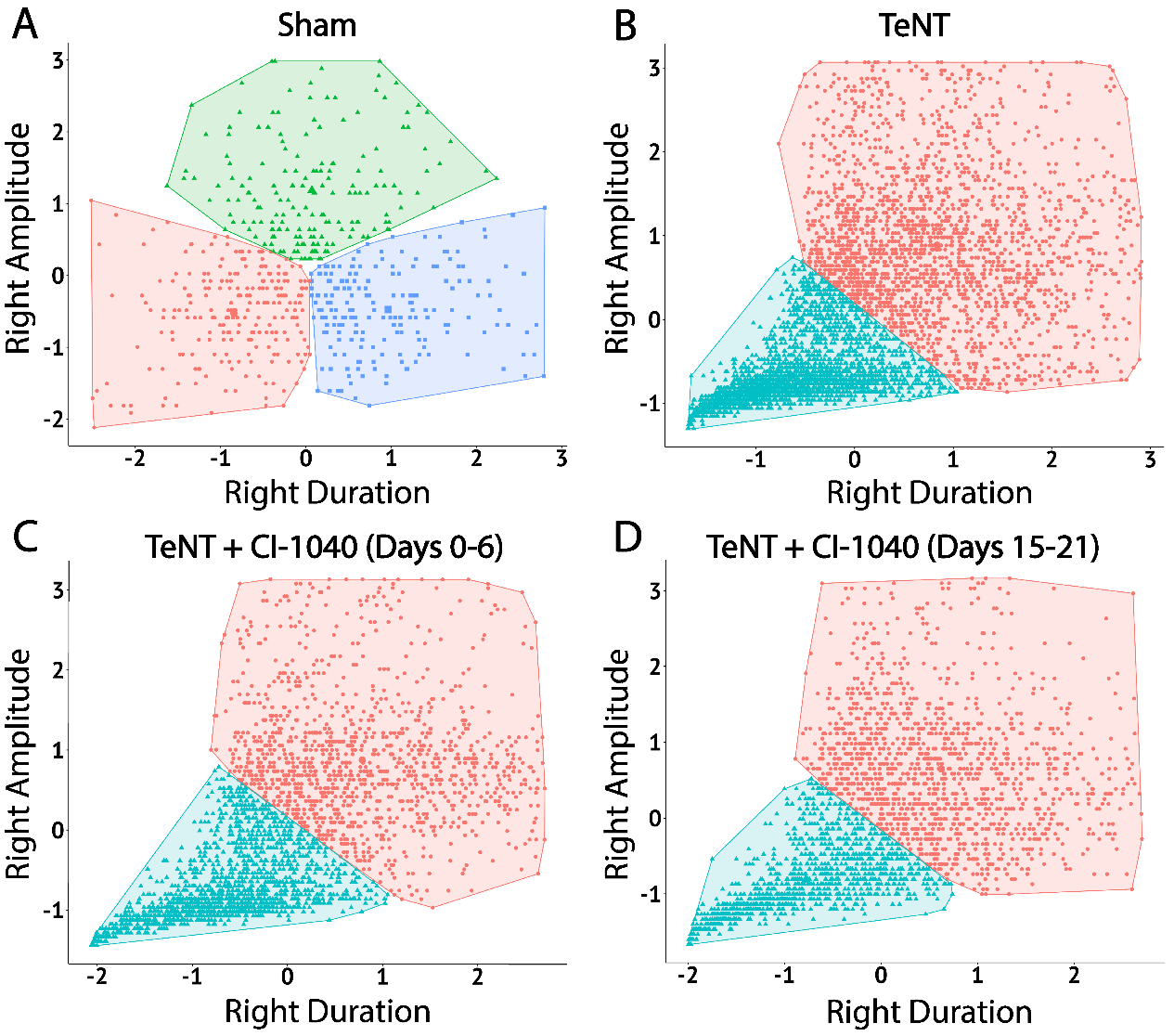


**Supplementary Figure 6 Example cluster analysis of interictal spikes based on morphologic parameters.** We performed a cluster analysis on all spikes observed at the toxin injection site (electrode L2) on post-operative day 49 for Sham, tetanus toxin (TeNT), and TeNT + CI-1040 groups. Each spike is visualized as a single datapoint on the scatterplot showing the relationship of the Right (second-half) Amplitude (y-axis) to the Right (second-half) Duration (x-axis). Amplitude and duration values are displayed as z-scores. Different colors indicate distinct clusters of spikes determined independently for each experimental group via k-means clustering. Separation among clusters is also indicated by the convex polygons drawn around adjacent sets of points. **(A)** Spikes in Sham animals fell into three distinct clusters with a broad spread of Right Amplitude and Right Duration, representing a unique distribution of spike morphologies compared to those observed in TeNT animals with or without drug treatment. **(B-D)** Spikes in TeNT animals were distributed into two distinct clusters and were more concentrated in the upper right quadrant of the scatterplots, corresponding to higher values both Right Amplitude and Right Duration (Right Slope).

**Supplementary Table 1** **Patient demographics for high- and low-spiking tissue samples**

| **Sample ID** | **Age** | **Sex** | **Relative Spike Activity** | **Spike Frequency (Mean Spikes/10 min)** |
| --- | --- | --- | --- | --- |
| 1 | 32 | F | Low | 0 |
| 2 | 15 | F | Low | 3 |
| 3 | 6 | M | Low | 0 |
| 4a | 7 | F | Low | 2 |
| 4b | 7 | F | High | 143 |
| 4c | 7 | F | High | 25 |
| 5a | 33 | M | Low | 62 |
| 5b | 33 | M | Low | 6 |
| 6a | 2 | M | High | 117 |
| 6b | 2 | M | High | 63 |
| 6c | 2 | M | Low | 0 |
| 6d | 2 | M | Low | 0 |
| 7 | 6 | M | Low | 7 |
| 8 | 1 | M | Low | 1 |
| 9a | 2 | F | High | 68 |
| 9b | 2 | F | High | 47 |
| 10a | 56 | F | Low | 51 |
| 10b | 56 | F | High | 299 |
| 11a | 11 | F | Low | 2 |
| 11b | 11 | F | High | 66 |
| 12a | 2 | M | High | 63 |
| 12b | 2 | M | High | 51 |
| 13 | 16 | M | High | 98 |
| 14a | 15 | F | High | 41 |
| 14b | 15 | F | High | 41 |
| 14c | 15 | F | High | 33 |
| 15a | 3 | M | High | 83 |
| 15b | 3 | M | High | 99 |
| 16 | 3 | F | High | 108 |

For human tissue analysis, we utilized 29 samples collected from 16 unique patients. Patients ranged in age from 1-56 years old. All patients were diagnosed with neocortical epilepsy. Samples selected for histological analysis had grossly normal pathology. Spike frequency for each region was calculated as the mean number of spikes per 10 minutes, averaged from three independent ten-minute recordings. High-and low-spiking regions are relative to each other from within the same patient, since each patient is internally controlled with two or more samples at the time of tissue collection.
